# Cold Atmospheric Plasma-Activated Water Irrigation Induces Defense Hormone and Gene expression in Tomato seedlings

**DOI:** 10.1038/s41598-019-52646-z

**Published:** 2019-11-06

**Authors:** Bhawana Adhikari, Manish Adhikari, Bhagirath Ghimire, Gyungsoon Park, Eun Ha Choi

**Affiliations:** Plasma Bioscience Research Center, Applied Plasma Medicine Center, Department of Electrical and Biological Physics, Kwangwoon University, Seoul, South Korea

**Keywords:** Plant molecular biology, Plant physiology

## Abstract

Plants are very vulnerable to pathogen attacks and environmental stress as they are exposed to harsh environments in natural conditions. However, they have evolved a self-defense system whereby reactive oxygen and nitrogen species (RONS) act as double-edged swords by imposing (at higher concentration) and mitigating (at lower concentration) environmental stress. Cold plasma is emerging as a feasible option to produce a variety of RONS in a controlled manner when amalgamate with water. Cold plasma activated/treated water (PAW) contains a variety of RONS at concentrations, which may help to activate the plant’s defense system components. In the present study, we examine the effect of cold atmospheric-air jet plasma exposure (15 min, 30 min, and 60 min) on the water’s RONS level, as well as the impact of PAW irrigation, (assigned as 15PAW, 30PAW, and 60PAW) on tomato seedlings growth and defense response. We found that PAW irrigation (priming) upregulate seedlings growth, endogenous RONS, defense hormone (salicylic acid and jasmonic acid), and expression of key pathogenesis related (PR) gene. 30 min PAW contains RONS at concentrations which can induce non-toxic signaling. The present study suggests that PAW irrigation can be beneficial for agriculture as it modulates plant growth as well as immune response components.

## Introduction

Plants, which are immobilized organisms, often face many challenges like harsh environments, pathogens and herbivores attacks. In response to abiotic and biotic stresses, plants have evolved an adaptive defense system for survival^1^. Reactive oxygen and nitrogen species (RONS) are byproducts of cellular metabolism and play a binary role in aerobic life, depending on their concentration^2^. RONS in small concentrations act as signaling molecules and regulate cellular growth, development, and defense processes. However, at very high concentrations they are deleterious to cellular biomolecules and lead to cell death^3^. Hydrogen peroxide (H_2_O_2_) and nitric oxide (NO) are the most studied and well known RONS in aerobic systems. H_2_O_2_ is the most stable and strong oxidant molecule and initiates the production of various other reactive oxygen radicals, such as superoxide, hydroxyl, and NO_x_, in cells. H_2_O_2_ predominantly generates as a byproduct of photorespiration and photosynthesis in chloroplasts and mitochondria. The H_2_O_2_ acts as a vital signaling molecule and stimulates the production of other signaling molecules such as Ca^2+^, mitogen activated protein kinase (MAPK), hormone-like salicylic acid (SA), jasmonic acid (JA), abscisic acid, and ethylene^4^. NO is another interesting signaling molecule, which has attracted a lot of attention due to its multifarious role in plant growth and development. NO transduces the environmental signals by cAMP, altering the Ca^2+^ concentration in the cytosol, and interacts with H_2_O_2_. NO regulates the gene expression of various antioxidant enzymes, pathogenesis related (PR) proteins, and MAPK^5^. H_2_O_2_ and NO signaling molecules exhibit a cross-talk and both synergistic and antagonistic interactions with each other^6^.

Both biotic and abiotic stresses cause RONS bursts, which induce the production of secondary metabolites as well as defense hormones precursors in plant cells. When a pathogen invades the host system, the plant endogenous RONS level increase, which triggers defense genes expressions, such as SA and JA synthesis pathway genes and PR genes. These genes kill the pathogen and prevent it from infecting other cells by the hypersensitive response and systemic acquired resistance (SAR)^7^. H_2_O_2_ and NO mutually modulate phytohormone synthesis in plant cells and activate SAR by inducing the concentration of SA, JA, ethylene, and brassinosteroid^8,9^.

The JA synthesizes in plant cells in response to wounding, herbivorous attacks, and necrotropic pathogen infections. It also mediates RONS bursts and activates the hypersensitive response^10^. JA levels are modulated via NO concentration in plant cells. Bioinformatics analysis of NO responsive genes and promoters revealed that JA synthesizes genes such as oxophytodienoic acid reductase and upregulates expression in response to NO elicitors in *Arabidopsis thaliana*^9^. SA is a derivative of phenol synthesized from chorismate by isochorismate synthase. It is amalgamated in response to pathogen infections and develops local and systematic resistance^11^. As well as JA, plants have another defense phytohormone, synthesized via octadecanoid pathways using linolenic acid as a precursor. JA promotes the synthesis of flavonoids and other phenolic compounds^12^. SA and JA both regulate plant vegetative growth, seed germination, root growth, nutrient uptake, water relations, Ribulose 1,5-bisphosphate carboxylase oxygenase (RuBisCO) activity, chlorophyll content, and stomata closure^13,14^. They also affect the redox homeostasis by regulating the activities of antioxidant enzymes such as peroxidase, polyphenol oxidase, superoxide dismutase (SOD), catalase (CAT), and phenylalanine ammonia lyase (PAL)^15,16^. The upregulation of SA and JA in plants can enhance both their pathogen resistance potential and their growth^13^.

Genetic engineering approaches have frequently been attempted to improve plant growth and defense quality^17,18^. However, concerns for genetically modified organisms and their products have limited the general use of this technology^19^. Hence in the present scenario, the priming of plants is most extensive research topic in area of plant protection. Priming is a physiological process to achieve the readiness status of plants against biotic and abiotic stress^20^. Primed plants have shown a vigorous and faster response against stress^21^. Priming was done to boost plant immunity against pathogens and stress. Small biomolecule and stimuli used for induced immune response are called immune inducer. Various natural and chemical (an analog of the natural compound) inducers have been reported^22^. Cold plasma and cold plasma treated liquids are also used to induce plant growth.

Cold atmospheric pressure plasma (CAP) has attracted a lot of attention from plant biologists from the past few years due to its potential for enhancing plant growth and defense. CAP technology is a harmless, sensitive, ecofriendly and economical approach to improve plant health as well as crop production^23^. CAP is an ionized gas consisting of a mixture of radicals, UV rays, and electromagnetic waves, which initiates the production of various RONS in micro-environments^24,25^. CAP treatment to water modifies its chemical property by converting water into a RONS cocktail. RONS have a significant impact on plant metabolism and plant growth and development^2^. H_2_O_2_ and NO, small signaling biomolecules, are produced endogenously in different cellular metabolism processes and regulate plant responses to various abiotic and biotic stresses^26^.

The previously cited literature on the regulatory role of RONS (especially H_2_O_2_ and NO) in cellular metabolism, growth, and defense motivates us to investigate the impact of cold plasma generated H_2_O_2_ and NO in the water on plant defense transcripts and phytohormone. Therefore, in this study we investigate the effect of different cold PAW irrigations on the growth of seedlings, endogenous RONS concentration, oxidative toxicity, defense related (antioxidant, PR, and hormone) gene expression, and defense hormone content in seedlings. The results of this study indicate that PAW is acting as an inducer for growth as well as defense related components. To our best knowledge, it is the first study which shows that PAW treatment induces defense hormone production as well as other transcripts in seedlings and acts as an immune inducer. On the basis of the present study’s results, a schematic diagram was prepared to show the biochemical and molecular events occurring in plant cells in response to PAW-induced RONS burst.

## Results

**Figure 1:**
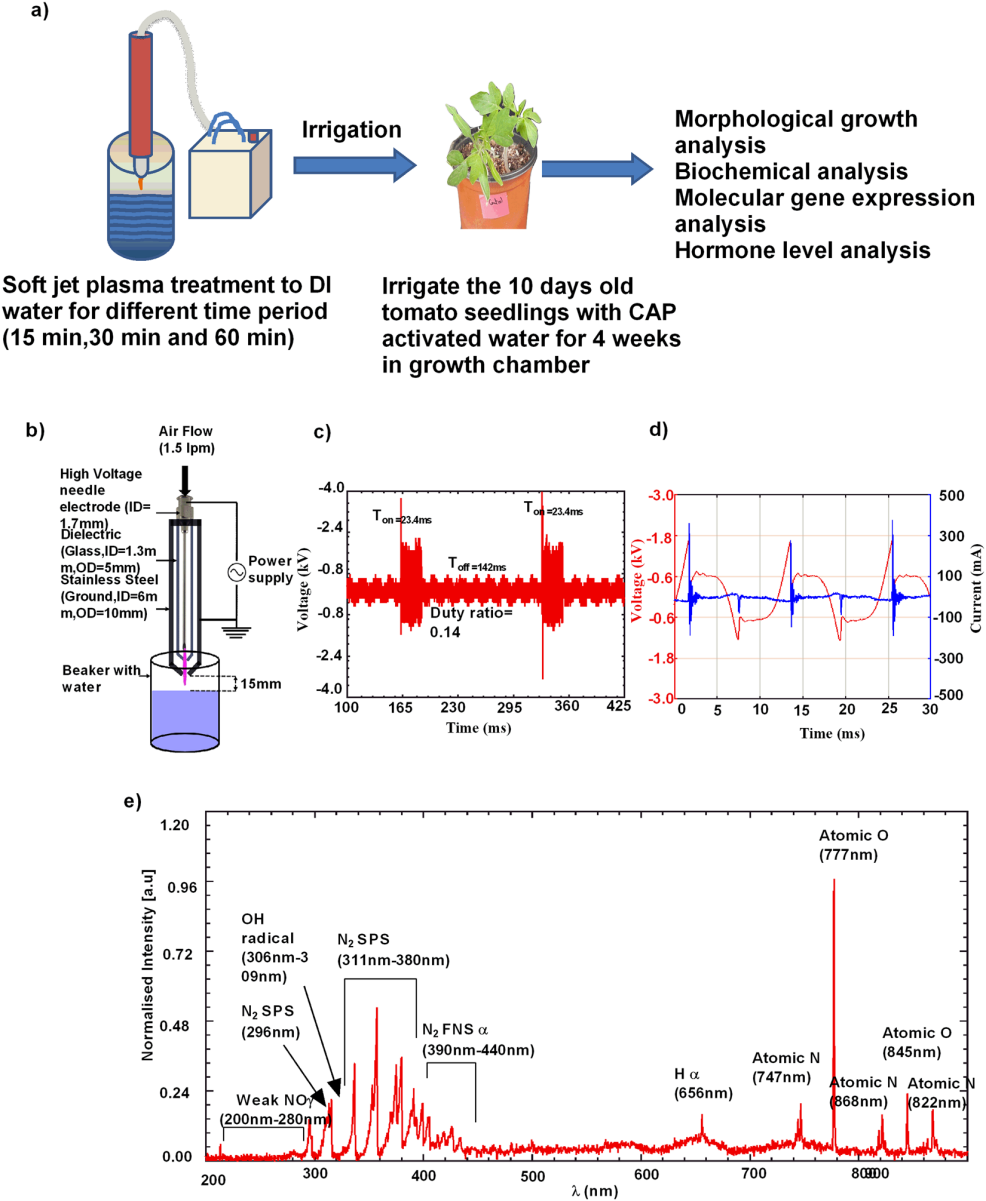
Experimental setup and physical characterization of the jet plasma. (**a**) Schematic representation of the work plan. (**b**) Schematic of the experimental setup. (**c**) Waveforms showing the on and off times of the plasma jet. (**d**) Current-voltage waveforms of the discharge during plasma on time. (**e**) Optical emission spectrum of the soft plasma jet with air gas from 200 to 900 nm.

We observed changes at physiological, biochemical and molecular level in tomato seedlings treated with PAW as compared to control seedling. The diagrammatic representation of the experiment strategy is shown in Fig. 1a. The air plasma jet instrument used in this study was developed in Plasma Research Bioscience Center, Kwangwoon University, Seoul, South Korea. The experimental setup of plasma treatment to water is shown in Fig. 1b. For irrigation, freshly prepared PAW was always used and all the physiological, molecular and biochemical parameter were assessed in 35 days old seedlings.

### Electrical and optical characteristics of plasma instruments

#### Electrical characteristics

The electrical characteristic of the discharge is shown in Fig. 1. The discharge was operated by a controlled circuit with on and off-times of 23.4 ms and 142.0 ms, respectively (Fig. 1c). The duty percentage for this condition is ~14%. A short operational time with long off-times was chosen to operate the discharge for a long time without overheating the electrodes. The current-voltage waveforms appearing during the on-time of the discharge period are shown in Fig. 1d. The voltage waveform appears distorted due to the discharge current peaks appearing in each half cycle of the applied voltage. A positive current peak appears with the increase of the applied voltage during the positive half cycle of the applied voltage. Some charges are accumulated within the dielectric material and are reversed back during the negative half cycle. As a result, a negative discharge current peak appears in the negative half cycle of the applied voltage. Here, the frequency, applied voltage, and current of the discharge are ~83.5 kHz, 0.66 kV, and 70.39 mA, respectively. The dissipated energy (P) per cycle was 3.57 J/s, which was obtained by integrating the current (I(t)) and voltage (V(t)) of one cycle (T) over the complete plasma on time^27^. Mathematically, P can be expressed as:

$$P={\int }_{0}^{T}I(t)V(t)dt$$

#### Optical characteristics

The normalized optical emission spectrum of the discharge for the air soft plasma jet is shown in Fig. 1(e). Weak emission signals from NOγ bands exist in the region between 200 and 280 nm. These species originate from the collision of energetic electrons with nitrogen and oxygen molecules present in the ambient air^28^. OH radicals are emitted at approximately 306–309 nm and originated from the collision of electrons, or metastable nitrogen atoms, with water molecules^27,29^. Strong emissions from various bands of the nitrogen second positive system (N_2_ SPS) are observed at 296 nm, 315 nm, 337 nm, 357 nm, and 380 nm.

Furthermore, there are emissions from the nitrogen first negative system (N_2_ FNS) between 390 and 440 nm, in addition to atomic nitrogen (742 nm, 822 nm, and 868 nm). The excited nitrogen species originate from the dissociation of nitrogen molecules present in the feeding gas and ambient environment. Strong emission from atomic oxygen (777 nm and 845 nm) is also observed, which is due to the dissociation of oxygen molecules. In addition to these species, emission from the hydrogen alpha line at 656 nm is also observed^27^.

### Chemical properties of water altered by the cold plasma exposure

**Figure 2:**
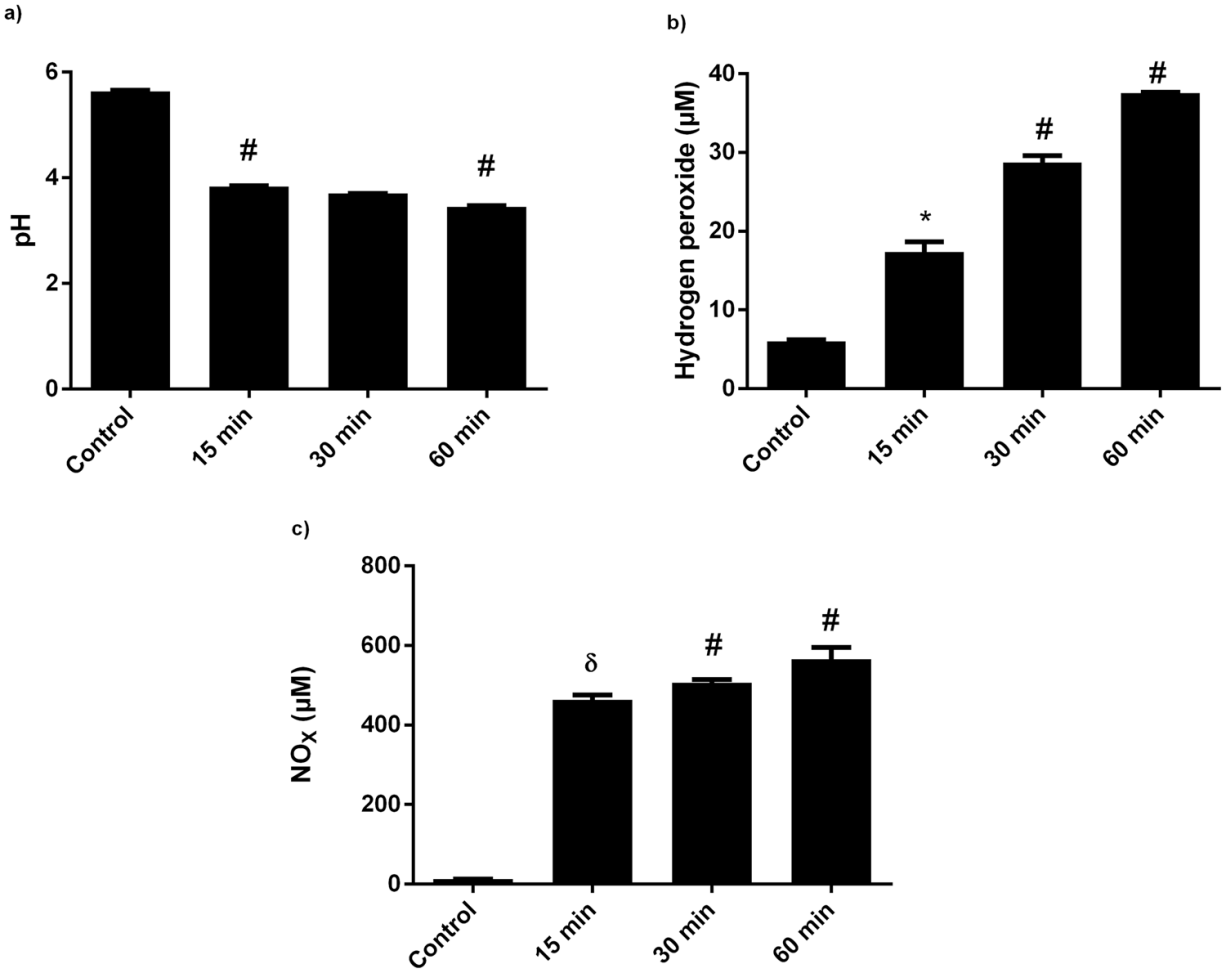
Biochemical properties of PAW. (**a**) pH, (**b**) H_2_O_2_, and (**c**) NO_x_ before and after different times of water exposure to plasma. Mean ± SE (n = 3) of each experiment represented in terms of error bars. The SE between the mean of the control and the treatment group was analyzed by student t-test. p-value denoted by ^*^ (p < 0.05), ^δ^ (p < 0.01), and ^#^ (p < 0.001).

The interaction of cold air plasma with water activates the production of different RONS in the water, which alters the chemical composition of water^29^. To determine the effect of CAP exposure on hydrogen ions concentration, the pH of water was measured at different time intervals after CAP exposure. The pH of water after 15 min CAP treatment was 3.8, which, compared to the control (pH of 5.6), is significantly lower. At 30 min and 60 min treatment, the pH of water decreased to 3.7 and 3.45, respectively (Fig. 2a). The CAP-induced changes in the chemical composition of the DI water were estimated by analyzing the H_2_O_2_ and NO amounts using a spectrophotometer. Compared to the control, the concentration of H_2_O_2_ increased significantly in all PAWs. The CAP exposure of 15 min, 30 min and 60 min to deionized water increased the H_2_O_2_ concentration 1.8-, 3.7-, and 5.25-fold, respectively (Fig. 2b). Similarly, the NO_x_ concentration also increased with increasing CAP exposure time. The NO_x_ concentration increased 35, 38, and 43-fold, compared to the control after the CAP treatment in water for 15 min, 30 min, and 60 min, respectively (Fig. 2c). The CAP produced various RONS in the gaseous phase, which, on interaction with water, produced long-lived RONS species and lowered the pH^30–32^.

### PAW irrigation induces growth of tomato seedlings

**Figure 3:**
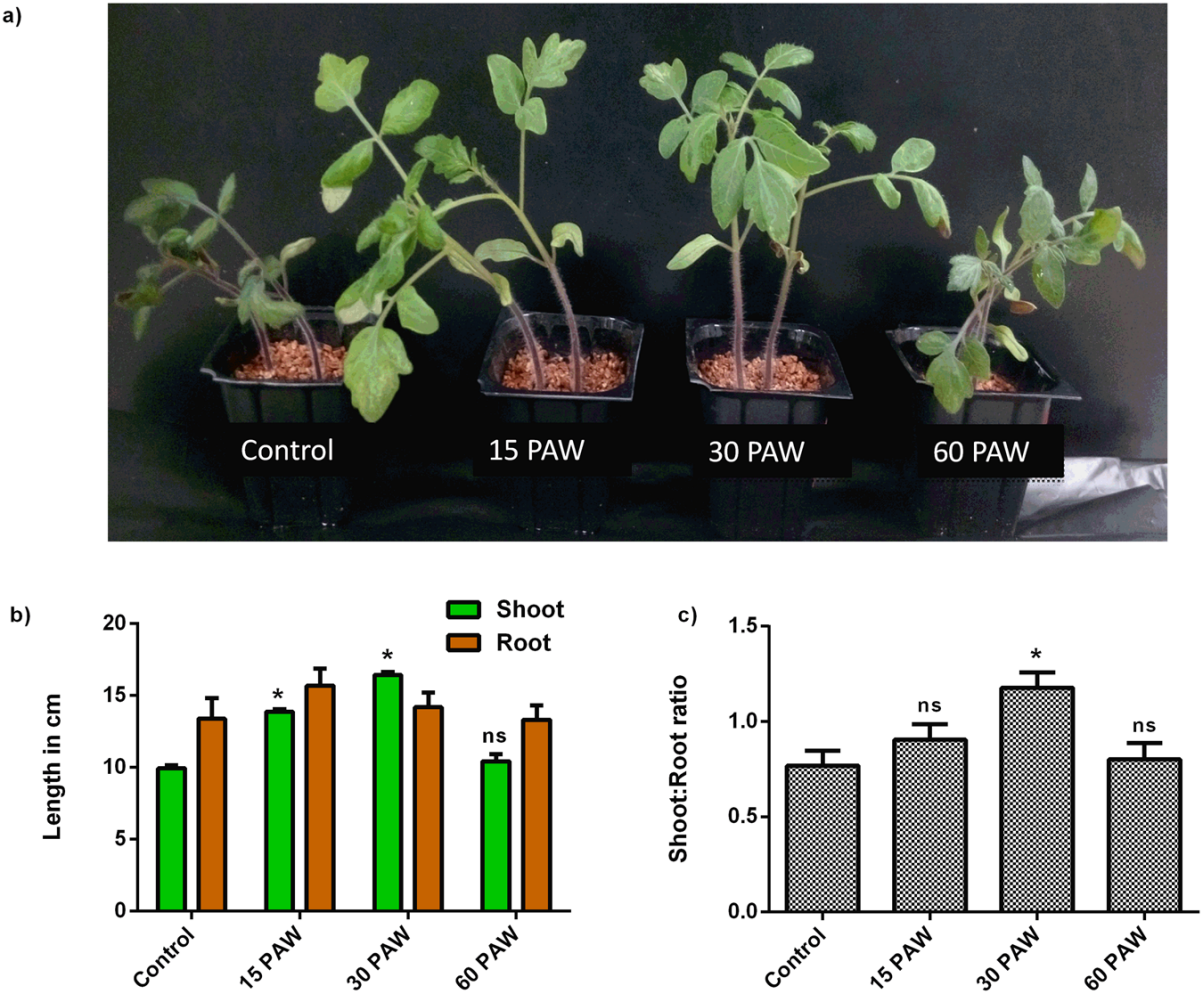
Effect of PAW treatment at different time-intervals on (**a**) physiological status of tomato seedlings (28 days old) grown in vermiculite. (**b**) shoot and root length (cm) and (**c**) shoot: root length ratio. ± SE of mean (n = 3) of each experiment represented in terms of error bar. Significant difference between the mean of control and treatment group was analyzed by student t-test. p-value denoted by ^*^ (p < 0.05), ^δ^ (p < 0.01), and ^#^ (p < 0.001).

The growth-inducing effect of PAW in seedlings was examined both morphologically and phenotypically. The tomato seedlings irrigated with 15PAW and 30PAW showed better morphological growth compared to the control seedlings (Fig. 3a). The phenotypically seedling growth was measured in terms of shoot and root lengths (Fig. 3b). Compared to the control samples, 15PAW and 30PAW-treated seedlings showed greater shoot and root length, whereas a non-significant change was observed in the 60PAW. The ratio of the shoot to root length maximum after the 30PAW treatments (Fig. 3c). These results indicate that PAW treatments enhance the growth of seedlings. Similar observations of the effect of PAW irrigation on lentil seedlings length was published by^33,34^. Previous publication^33^ reported that PAW-treated lentil seedlings exhibited 34% and 128% increase in the seedling length after the 3^rd^ and 6^th^ days of irrigation. Few reports also observed that the air PAW induced significant seedling growth compared to O_2_, N_2_, and He PAW^35^.

### Enhancement of endogenous RONS species by PAW

**Figure 4:**
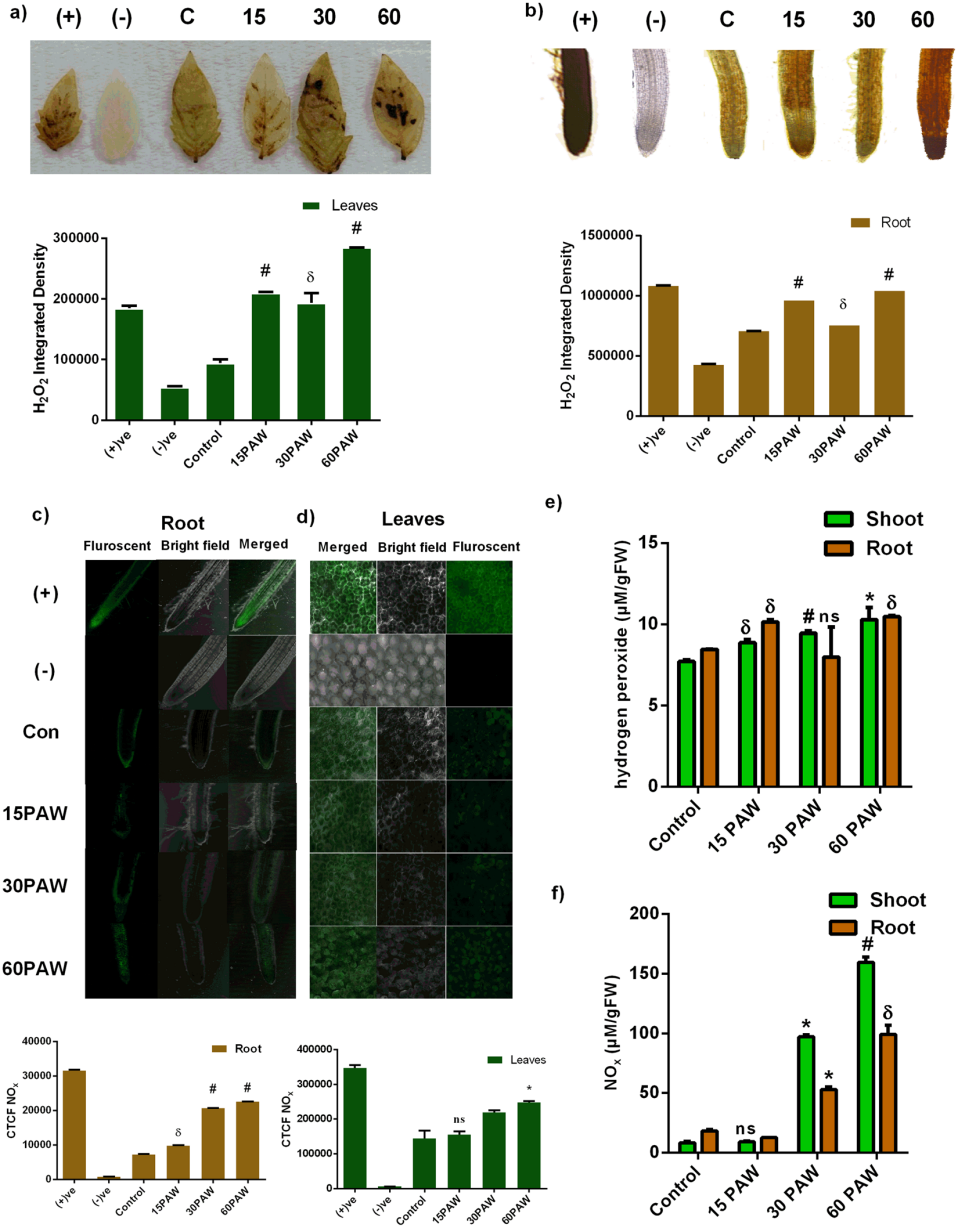
Biochemical representation of endogenous H_2_O_2_ in (**a**) leaves and (**b**) root tips. Microscopic NO_x_ detection in tomato seedlings (**c**), leaves, and (**d**) root tips. Spectrophotometric quantitation of (**e**) H_2_O_2_ and (f) NO (NO_x_) in seedlings. ±SE of mean (n = 3) of each experiment represented in terms of error bar. The significant difference between the mean of the control and the Paw-treated group was analyzed by student t-test. p-value denoted by ^*^ (p < 0.05), ^δ^ (p < 0.01), and ^#^ (p < 0.001).

To determine the effect of PAW treatments on the plant’s endogenous RONS concentration, H_2_O_2_ and NO_x_ were directly chemically detected in leaves and roots (Fig. 4). *In vivo*, H_2_O_2_ reacts with DAB and produces brown precipitates whose intensities were measured by ImageJ software and were used to prepare a graph. *In vivo* examination of H_2_O_2_ in leaves showed that, compared to the control samples, the H_2_O_2_ stain area increases in all PAW-treated plants (Fig. 4a). The spot intensity of 15PAW, 30PAW, and 60PAW seedlings leaves significantly increased compared to the control. Similarly, the PAW-treated roots had a higher H_2_O_2_ stain intensity compared to non-PAW-treated plants (Fig. 4b).

*In vivo* RNS content was detected in leaves and roots of seedlings using a fluorescent dye 4-Amino-5-Methylamino-2′,7′-Difluorofluorescein Diacetate (DAF-FM Diacetate) and fluorescent images were captured by confocal microscopy. Corrected total cell fluorescence (CTCF) of RNS in leaves and roots was measured by ImageJ software. The CTCF of RNS increased significantly in the root of all PAW-treated tomato seedlings (Fig. 4c), whilst in leaves, a non-significant induction in CTCF values of RNS was observed in 15PAW and 30PAW seedlings. A significant alleviation in CTCF of RNS was observed in the 60PAW plants (Fig. 4d).

H_2_O_2_ plays a very crucial role in the cascade of the free-radical reaction mechanism. Hence, it was assessed in the shoot and roots of tomato seedlings. H_2_O_2_ concentration increased in 15PAW, 30PAW, and 60PAW seedlings. H_2_O_2_ levels in 15PAW, 30PAW, and 60PAW shoot increased 0.14-, 0.22-, and 0.32-fold, respectively, compared to the control (Fig. 4e). In seedling roots, 15PAW and 60PAW exhibited similar patterns of H_2_O_2_ induction (0.19 fold and 0.23 fold), whereas 30PAW roots had no significant difference in H_2_O_2_ concentration compared to control seedlings (Fig. 4e).

The RONS species generated in water by plasma treatment also enhanced the endogenous level of NO_x_ in plants. In 30PAW and 60PAW seedlings, the shoot showed a significant improvement (10.7 fold and 18.3 fold) in NO_x_ concentration compared to the control, whereas the 15PAW showed a smaller increase in NO_x_ concentration. Similar upregulation of NO_x_ concentration was observed in PAW-treated tomato seedlings roots. In 30PAW and 60PAW seedlings roots, the NO_x_ level increased up to 1.9 and 4.4-fold, respectively, compared to the control (Fig. 4f). These results suggest that PAW-induced RONS positively influence the endogenous RONS status of seedlings.

### 15 min and 30 min PAW irrigation induce RONS signaling, not RONS toxicity, in tomato seedlings

**Figure 5:**
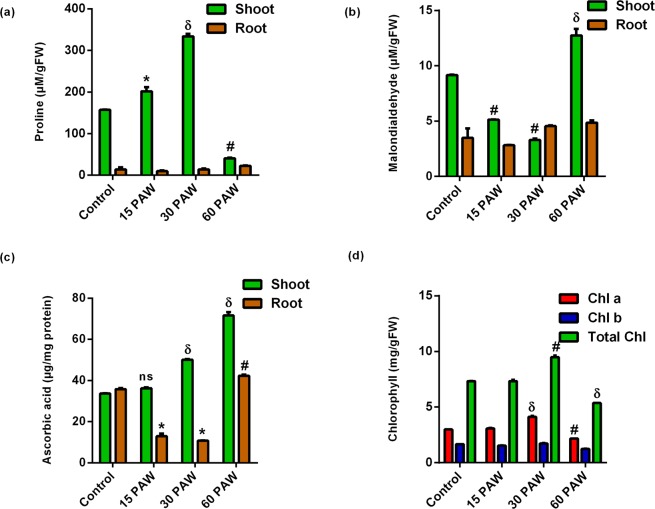
Biochemical status of tomato seedlings treated with 15PAW, 30PAW, and 60PAW. Content of (**a**) Proline, (**b**) Malondialdehyde (**c**) Ascorbic acid, and (**d**) chlorophyll in shoot and roots. ± SE of mean (n = 3) of each experiment represented in terms of error bar. The significant difference between the mean of the control and the treated group was analyzed by student t-test. p-value denoted by ^*^ (p < 0.05), ^δ^ (p < 0.01), and ^#^ (p < 0.001).

Several biochemical markers of stress were analyzed in shoots and roots of seedlings to measure the oxidative damage potential of PAW-induced RONS in the plant cells. Proline, an imino acid, acts as an osmolyte and redox buffer in cytoplasm^36^. Proline content of 15PAW and 30PAW shoots increased significantly but reduced in 60PAW shoots compared to control. In roots, no significant changes were observed. Compared to the control root, the proline content of 15PAW and 30PAW seedlings roots was slightly lower, but it was 0.5-fold higher in the 60PAW seedlings (Fig. 5a). Lipid Peroxidation is an indicator of oxidation of lipids membranes by RONS and, in this experiment, was detected by analyzing the accumulation of MDA, a byproduct of lipid oxidation reaction in plants. The MDA content of the 15PAW and 30PAW shoots was lower (0.44-fold and 0.64-fold) than in the control shoot. However, the MDA content of the 60PAW shoot increased 0.39-fold, compared to the control. In roots, the MDA content was lower in the 15PAW but was not significant in the 30PAW and 60PAW with respect to the control roots (Fig. 5b). Ascorbic acid levels were also determined in PAW-treated plant spectrophotometrically. 15PAW, 30PAW, and 60PAW leaves increased the ascorbic acid content by 0.07, 0.48, and 1.15-fold, respectively. However, a reduction in the ascorbate content was observed in the roots of 15PAW and 30PAW (Fig. 5c). The chlorophyll content is a measure of photosynthetic activity and its presence was enhanced in the 30PAW, while it was reduced in the 60PAW, compared to the control (Fig. 5d).

These results indicate that the 15PAW and 30PAW treatments do not induce any oxidative damage and the 30PAW irrigation improved the antioxidant potential and reduced oxidative toxicity. Longer exposures (60 min) of CAP to water produced excessive RONS in 60PAW, which initiated the RONS stress or toxicity. This suggests that shorter exposure of water to CAP (15 min and 30 min) produces amounts of RONS that activate plant growth but are not toxic.

### PAW up-regulated defense response and antioxidant gene expression

**Figure 6:**
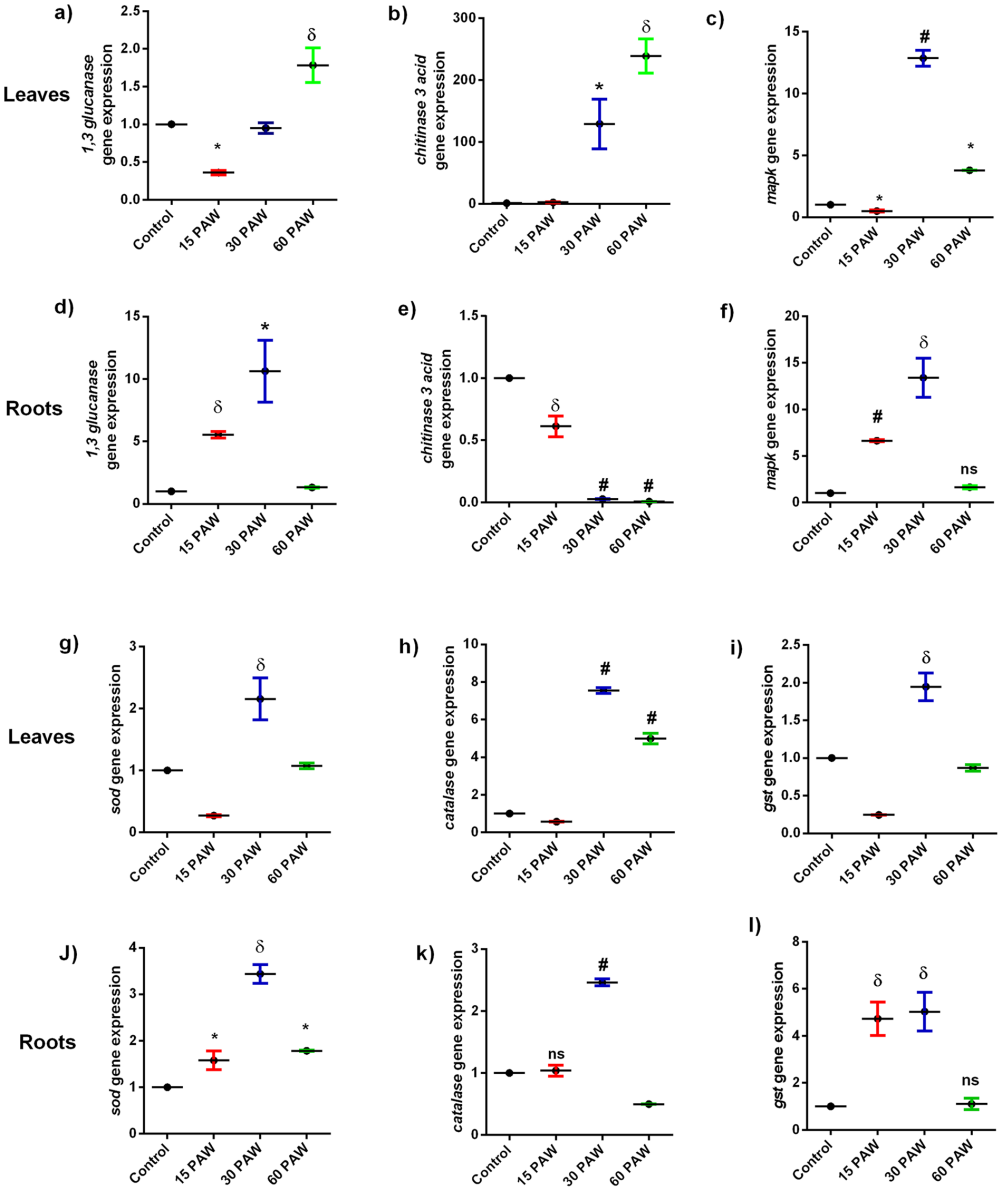
Quantitative PCR for gene expression analysis of different pathogenesis gene. β− 1–3 glucanase (**a**): leaves, (**d**): roots), Chitinase 3 acid (**b**): leaves, (**e**): roots), MAPK (**c**): leaves, (**f**): roots), and redox homeostasis genes, such as Superoxide dismutase (**g**): leaves, (**j**): roots), Catalase (**h**): leaves, (**k**): roots), Glutathione s transferase (**i**): leaves, (**l**): roots) in different PAW treatments in tomato seedlings. ± SE of mean (n = 3) of each experiment represented in terms of error bar. The significant difference between the mean of the control and the treated group was analyzed by student t-test. p-value denoted by ^*^ (p < 0.05), ^δ^ (p < 0.01), and ^#^ (p < 0.001).

To understand the effect of the PAW treatment on plant defense and antioxidant potential, the expression of various genes related to plant redox homeostasis and pathogenesis resistance were examined by qPCR. Pathogenesis related gene expression elicits in response to pathogen invasion in plant cells via MAPK signaling^37^. Chitinase 3 acidic and β−1, 3 glucanase is antifungal proteins that hydrolyze the cell wall of a pathogen. β −1, 3 glucanase is also involved in molecular trafficking through plasmodesmata and flower formation^38,39^. β−1, 3 glucanase gene expression was lower in 15PAW and 30PAW than in 60PAW leaves (Fig. 6a). However, 15PAW and 30PAW roots showed higher expression of β−1, 3 glucanase gene expression (Fig. 6d). Expression of chitinase 3 acidic gene significantly upregulated in leaves of 30PAW and 60PAW (Fig. 6b) but in the roots of PAW treated seedlings, its expression was reduced successively in 15PAW, 30PAW, and 60PAW compared to non-PAW treated seedlings (Fig. 6e). The MAPK responsible to transduce the signaling by phosphorylating the other signaling pathway proteins showed higher expression at 30PAW, while its expression level decreased in 60PAW seedlings leaves (Fig. 6c). The *mapk* gene expression in roots showed similar patterns to the leaves in which 30PAW showed a higher gene expression than the 15PAW treated plant roots, which again declined at 60PAW (Fig. 6f).

To maintain the redox homeostasis in plant cells, antioxidant enzymes are expressed continuously. The expression of antioxidant enzymes enhances as the level of RONS content is induced in biotic stress conditions^40^. SOD enzymes detoxify the superoxide radical and produce H_2_O_2_. The expression of the *sod* gene upregulated one fold at 30PAW leaves and 2.4-fold at 30PAW root, compared to the control seedlings. The SOD expression was lower in 15PAW leaves but induced a 0.5-fold increase in 15PAW roots. In 60PAW leaves, the *sod* gene expression of seedlings decreased significantly compared to the 30PAW and was comparable to the control, while in roots it was comparable to the 15PAW (Fig. 6g,j). The Catalase enzyme catalyzes the quenching of H_2_O_2_ during photorespiration and is responsible for H_2_O_2_ decomposition in plant cellular organelles, such as peroxisome and mitochondria^41^. Catalase expression was the highest in 30PAW seedlings and it was 6.5-fold in leaves and 1.4-fold in roots, compared to the control (Fig. 6h,k).

In the 60PAW seedlings, *cat* expression increased 3.9-fold in leaves but decreased significantly in roots compared to the control. Glutathione S transferase also quenches RONS and protects the plants from oxidative stress. Glutathione S transferase (*gst*) expression was upregulated in 30PAW leaves (0.9-fold) but decreased in seedlings leaves treated with 60PAW. In 15PAW roots and 30PAW seedlings, gene expression induced a 3.7-fold and 4-fold, respectively (Fig. 6I,l).

### PAW treatment enhances SA and JA content in tomato seedlings

**Figure 7:**
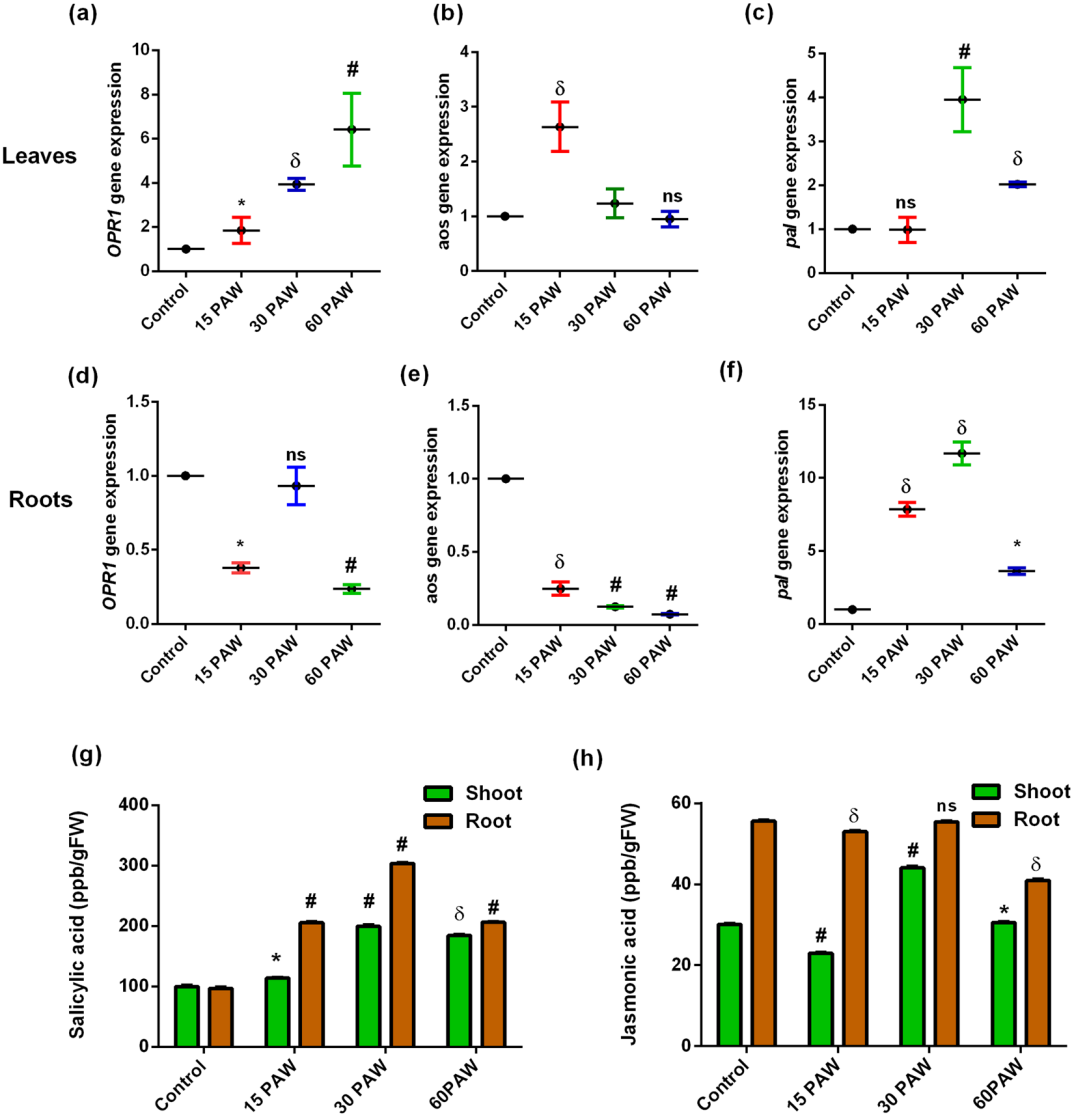
SA and JA status in PAW treated tomato seedlings. Gene expression analysis of key enzymes of JA and SA synthesis pathways. 12-Oxophytodienoic acid reductase (opr 1) (**a**): leaves, d: roots), Allene oxidase synthase (aos) (**b**): leaves, (**e**): roots) and Phenylalanine ammonia lyase (pal) (**c**): leaves, f: roots). LC-MS analysis of SA and JA content. (**g**) SA content in leaves and root and (**d**) JA content in leaves and roots of tomato seedlings. ± SE of mean (n = 3) of each experiment represented in terms of error bar. The significant difference between the mean of the control and the treatment group was analyzed by student t-test. p-value denoted by ^*^ (p < 0.05), ^δ^ (p < 0.01), and ^#^ (p < 0.001).

Phytohormones regulate various developmental, metabolic, and defense mechanisms of plants^42^. SA and JA are phytohormones synthesized in response to biotic stress, such as pathogens and herbivores attacks. 12-Oxophytodienoic acid reductase (*opr 1*) and allene oxidase synthase (*aos*) are regulatory genes of JA synthesis^43^. The mRNA level of *opr1* and *aox* were induced in leaves by the PAW treatment. The expression of *opr1* gene induces in 15PAW, 30PAW, and 60PAW leaves compared to the control (Fig. 7a). In the roots of the PAW treated seedlings, gene expression significantly reduced as a result of the 15PAW and 30PAW treatments with respect to control (Fig. 7d). Allene oxidase synthase (*aos*) gene expression level was the highest in the 15PAW leaves and was reduced in the roots of all PAW treatments (Fig. 7b,e). In the 30PAW and 60PAW leaves, no significant difference in aos mRNA profiling was detected. Phenylalanine ammonia Lyase (PAL) catalyzes the first step of SA and other phenolic compound synthesis^44^. Phenylalanine ammonia lyase expression was maximum in the 30PAW (2.9-fold) followed by the 60PAW leaves (1.0-fold) (Fig. 7c). Similarly, the highest *pal* gene expression (10.6-fold) was observed in the 30PAW followed by the 15PAW and 60PAW roots (6.8-fold and 2.6-fold, respectively) compared to control seedlings roots (Fig. 7f).

LC-MS is a fast and sensitive method to detect the phytohormone content in plant extracts^45^. Therefore, LC-MS of PAW treated seedlings (leaves and roots) was performed to validate the qPCR results. A standard graph for both hormones, SA and JA, was prepared using various concentrations (5 ppb, 10 ppb, 20 ppb, 50 ppb, 10 ppb, 500 ppb, and 1000 ppb) of the standard solution. SA showed the peak at retention time: 6.36 min and JA peak confirmed at RT: 7.42 min in the chromatogram (Supplemental figure S2). The SA content was upregulated in all PAW treatments and was maximum in 30PAW seedlings leaves (0.99-fold) and roots (2.14-fold) (Fig. 7g). JA followed a similar pattern of SA in the PAW treated seedlings leaves. In the 30PAW leaves, the JA content induced the highest (0.44-fold) as compared to control. On the other hand, no significant effect on the JA content of the 15PAW and 30PAW seedling roots was observed, while in the 60PAW the JA concentration was lower than in the control plant (Fig. 7h). AOS and OPR1 are two key enzymes of the JA biosynthesis pathway. Both of these enzymes expression affects the JA biosynthesis. In the present study, 30PAW irrigation induced expression of AOS and OPR1 gene in leaves which mutually leads to the induction in JA. Although, 15PAW and 60PAW showed only significant induction either in AOS or OPR1 gene hence less regulating effect on JA biosynthesis and content. Similarly in the case of roots, lower expression of AOS and OPR1 enzyme which leads to a significant and non-significant reduction in JA content.

All these results indicate that the irrigation of tomato seedlings with PAW leads to many physiological, biochemical, molecular, and hormonal alterations. The 15PAW treatment showed a positive stimulatory effect on plant growth. The 30PAW treated seedlings showed better growth, antioxidant, hormone status, and defensive gene expression than all other PAW-irrigated and control seedlings. The 60PAW treated seedlings had higher antioxidant and PR gene expression with the accumulation of higher RONS, which resulted in more lipid peroxidation and chlorophyll degradation.

## Discussions

The present study was designed to investigate the effect of PAW irrigation on the plant defense system. This study also links the RONS generated by CAP treatment to the modulation in plant defense system. The choice of feeder gas plays a vital role in generating the diversity of RONS in CAP. It is already documented that the use of air as feeder gas in CAP produces a greater variety of RONS than other gases, such as argon, N_2,_ and O_2_^35^. Here, the optical emission spectra of air jet CAP revealed the presence of various peaks of hydroxyl radicals, nitric oxide, atomic oxygen, atomic nitrogen, and reactive nitrogen molecule species. These species interact with water molecules and contribute to the alteration of the physicochemical properties of water. Interaction of water with these CAP generated reactive hydroxyl radicals, atomic oxygen, nitrogen, and active molecule nitrogen species leads to the production of other RONS, such as H_2_O_2_ and NO_x_. H_2_O_2_, NO, nitrate ions, and peroxynitrous acid are predominantly produced within PAW and contribute to acidifying water. Although in the present study we focus mainly on the presence of H_2_O_2_ and NO_x_ in PAW, other species were observed in PAW by^33^.

These RONS contribute to both reducing the pH of the PAW and modifying its chemical composition, hence affecting the plant growth. Sivachandiran^46^ also reported a similar decrease in pH of water on plasma treatment and its seedling growth-inducing effect in radish (*Raphanus sativus*), tomato (*Solanum lycopersicum*), and sweet pepper (*Capsicum annum*). The induced concentration of NOx in PAW was expected to have a growth-inducing impact on the seedlings. Previous reports also suggest that nitrite and nitrate ions in PAW act as nitrogen fertilizers and contribute to the enhancement of seedlings growth^47^. OES spectrum of air jet plasma showed the presence of atomic oxygen which can contribute in the generation of molecular oxygen in PAW. Molecular oxygen is a well-known product of chemical reactions occurring in PAW^30^. Papadopoulos^48^ reported that molecular oxygen improved the nutrition absorption efficiency of roots. The present investigation suggested that another possible explanation of this growth enhancement is the higher oxygen content of PAW, which improves the nutrition uptake capacity of roots. Thus, both nitrate ions and molecular oxygen are required to induce the seedling growth.

In the present study, PAW irrigation enhanced endogenous H_2_O_2_ and NO_x_ levels in seedlings. Hence, RONS present in plasma treated water influence the endogenous RONS level. These observations were supported by Mejia-Teniente, 2013^49^ study, where the exogenous application of 14 mM and 18 mM H_2_O_2_ solution induced the endogenous H_2_O_2_ content of *Capsicum annuum* plant. The exposure time of the plasma treatment of water plays an important role in regulating the concentration of RONS in water and plants. Shorter exposure to plasma, such as 15 min and 30 min, produced sufficient RONS to induce RONS signaling in seedlings. At the basal level, H_2_O_2_ and NO RONS act as signaling molecules in plants^2^. Longer exposure to plasma (60 min) generates excessive RONS and initiates the oxidative stress in the seedlings. Hence, 15PAW and 30PAW seedlings have a better biochemical stress and growth profile than the control and the 60PAW. The RONS in PAW-treated seedlings initiate RONS signaling and gene expression, and lead other biochemical changes in the plant cell. In previous studies, the pretreatment of H_2_O_2_ solution to soybeans seedling enhanced the endogenous H_2_O_2_ content and increased the chlorophyll content and lipid peroxidation of soybean leaves^50^. Jiang^51^ reported higher accumulation of H_2_O_2_ and antioxidants (peroxidase and polyphenol oxidase) activity in plasma treated tomato seedlings. Likewise^34,52^ reported that seeds exposure to cold plasma exposure induce proline, total soluble sugar content, antioxidant genes, PR genes, and reduce the MDA content in seedlings. Therefore, it is proposed that H_2_O_2_ and NO_x_ present in PAW and exogenously applied H_2_O_2_ and NO_x_ solution may follow similar mechanisms of action and have a similar impact on plant cells.

Transcription regulation studies of H_2_O_2_ and NO in tobacco plants revealed that H_2_O_2_ and NO mutually regulate expression of defense related genes^49,53^. In the present investigation, we observed that mitogen activated protein kinase (*mapk)* gene expression was induced in both root and shoot of PAW treated tomato seedling. Capone^50,54^ found that MAPK induction in the shoot in response to H_2_O_2_ and NO was similar in both conditions when applied through root or direct injection to leaves and proposed that the transmission of exogenous H_2_O_2_ and NO signals from root and shoots. From the present study, as well as previous reports, it is clear that H_2_O_2_ and NO both induce the MAPK signaling pathway and regulate the expression of defense related genes. Higher expression of defense related gene expression was observed in leaves of PAW treated seedlings^55^. Here, a diverse profile of biochemical markers and gene expression was observed in roots and leaves of PAW-treated seedlings. Earlier studies on plant immune response reported similar tissue specific differential immune responses of roots and suggested the compartmentalization of defense mechanisms in root systems^56^. Panngom^57^, observed that direct treatment of cold plasma to tomato leaves induces the gene expression in the roots only. Therefore, in light of the present observations and earlier reports we propose that RONS transmit from the site of exposure to other parts of the plant and are differentially regulated at the molecular and biochemical levels.

Plants synthesize various phytohormones in response to biotic stress which upregulates the production of small defense proteins and strengthens the plant’s immune system. SA and JA are key regulatory immune phytohormones^13^. When a pathogen invades the host system, it firstly induces endogenous RONS levels, which trigger hormone synthesis regulatory genes expression, defensive protein, and PR genes synthesis. These synthesized genes kill the pathogen and prevent it from infecting other cells by hypersensitive responses and SAR^7^. In the present investigation, PAW irrigation stimulated SA and JA synthesis pathway enzymes expression and increased their content in plant leaves and roots. H_2_O_2_ and NO are the primary reactive species generated in seedlings by PAW irrigation. H_2_O_2_ triggered the expression of SA synthesis pathway genes and its accumulation in cell^58^. SA induced PR gene expression and led to SAR. NO induced the expression of pathogenesis related proteins, antioxidant enzymes, root growth, and JA synthesis^59^. In *Arabidopsis thaliana*, JA synthesis pathway genes expression was upregulated in response to NO content^9^. Here, we proposed that PAW-mediated H_2_O_2_ and NO_x_ induction in plants mimics the RONS burst via pathogen attack and exhibit changes in gene expression.

**Figure 8:**
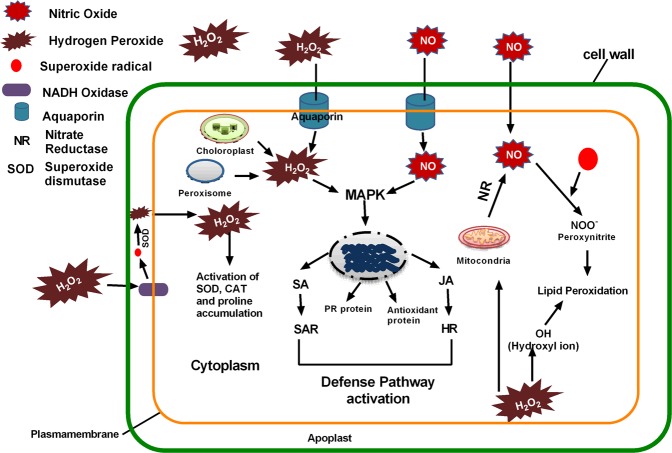
Schematic representation of PAW generated RONS and RNS effects on plant cellular metabolism. PAW H_2_O_2_ and NO either transport via aquaporin or induce the production of RONS in cytoplasm via NADH oxidase. H_2_O_2_ and NO initiate the production of other RONS in cytoplasm and enhance intracellular RONS concentration. These RONS activate defense pathway genes by inducing PR gene expression, antioxidant gene expression, SA, and JA content.

In summary, all the biochemical events and modifications by RONS (H_2_O_2_ and NO) in plant cells are demonstrated in Fig. 8. Cold plasma treated-water irrigation induced H_2_O_2_ and NO levels increase in the extracellular space. From the extracellular space H_2_O_2_ and NO either directly transport into cell cytoplasm through aquaporin or induce the production of H_2_O_2_ from NADH oxidase, chloroplasts, peroxisome, and NO from mitochondria. H_2_O_2_ and NO both activate the production of other RONS, such as superoxide ion, peroxynitrite, and hydroxyl radicals in the cytoplasm, which causes lipid oxidation at higher concentrations. H_2_O_2_ and NO both induce the expression of PR proteins, antioxidant enzyme activity, SA and JA pathway enzymes via MAPK signaling pathways. All these defense proteins and phytohormones strengthen the plant pathogen defense pathway.

Overall, the present study suggests that PAW can act as both plant growth inducer as well as immune inducer. Therefore, in the future, studies on cold plasma generated RONS signaling in plants need to be emphasized for a better understanding of the potential of cold plasma in plant growth, development, and defense.

## Materials and Methods

### Characterization of the CAP device and water treatment

A schematic of the experimental setup is shown in Fig. 1(a). The plasma jet consists of a high voltage needle electrode (ID = 1.7 mm), which is inserted inside a glass dielectric tube (ID = 3.3 mm, OD = 5 mm). The glass tube is inserted inside a cylindrical stainless-steel metal electrode (ID = 6 mm and OD = 10 mm), which is connected to the ground. The distance from the end of the dielectric glass to the end of the grounded metal electrode is 1 mm. Air gas at a flow rate of 1.5 liters per minute is used through the inner needle electrode as a feeding gas. Discharge occurs when the alternating voltage is applied between the power needle electrode and the outer grounded electrode. To produce the PAW, a 50-ml beaker is filled with de-ionized (DI) water and the distance from the end of the grounded electrode to the top of the water surface is kept at 15 mm (Fig. 1a) and treated at the following exposure times: 15 min, 30 min, and 60 min.

### Assessment of plant growth

Tomato (*Solanum lycopersicum*) was used to investigate the effect of PAW on the phytohormone status. Tomato (cultivar named “titichal”) seeds were obtained from Nongwoo Bio, Suwon, South Korea. They were first germinated in petriplates on wet paper towels and left in darkness at room temperature for two days. The germinated tomato seeds were then transferred into a vermiculite pot and grown in a growth chamber under controlled conditions (temp: 22 ^ο^C, humidity: 60%, photoperiod: 16/8 hours light/dark). After ten days of germination, the tomato seedlings were irrigated with 10 ml of PAW, whilst control seedlings were irrigated with DI water. Plasma-activated water treatments were performed two times per week for up to five weeks. Subsequently, after the completion of the treatment, the five weeks old (35 days) tomato seedlings were used for physiological, biochemical, molecular, and phytohormone analysis (Supplemental figure S1). Seedlings irrigated with PAW with 15 min, 30 min and 60 min treated plasma activated water are referred to as 15PAW, 30PAW, and 60PAW, respectively.

### Estimation of H_2_O_2_ and NO_x_

Endogenous H_2_O_2_ of tomato seedlings and PAW were detected spectrophotometrically using QuantiChrom^TM^ Peroxide assay kit (Bioassay Systems, USA). In this assay, a purple color complex was formed after oxidation of Fe^2+^ into Fe^3+^ by H_2_O_2_ (Fe^3+^-xylenol orange reaction), which was measured at 540–610 nm. *In vivo* visualization of H_2_O_2_ in tomato leaves and roots was carried out using 3,3-diaminobenzidine (DAB) as substrate^60^. Firstly, leaves and roots were carefully excised from seedlings and immersed in DAB solution (1 mg/ml) under darkness for eight hours. DAB reacted with H_2_O_2_, resulting in a brown-colored product in seedlings leaves and roots. Next, for better visualization of the brown spots, chlorophyll was removed by immersing leaves in ethanol (96%) and keeping them at 65 °C in an incubator for one hour. Finally, leaves and roots were washed twice with ethanol and were photographed. Prior to DAB staining, positive control leaves and roots were incubated with 10 mM H_2_O_2_ for 10 min. Microscopic images were analyzed by ImageJ software (https://imagej.nih.gov) to determine the respective DAB staining intensities.

The NO_x_ amount was measured in plants and PAW by QuantiChrom^TM^ NO assay kit (Bioassay Systems, USA)^61^. The NO level was measured by the reduction of nitrate to nitrite using the Griess method at 540 nm spectrophotometry. Endogenous detection of reactive nitrogen species RNS in seedlings roots and leaflets were performed by the DAF-FM Diacetate (4-Amino-5-methylamino-2,7-Difluorofluorescein Diacetate) (ThermoFisher Scientific, USA) fluorescence dye using an Olympus IX83-FP confocal microscope (Olympus, Japan)^62^. For endogenous detection of NO, plant roots and leaves were excised and washed with DI water. Five-mm root tips were cut and the leaf abaxial epidermis was peeled before immersing it in 10 µM of DAF-2DA (prepared in 10 mM MES-KCl buffer, pH 7) followed by 5 min incubation in a vacuum chamber and 15 min at room temperature in a dark chamber. After incubation, plant samples were thoroughly washed with 10 mM MES-KCl buffer and examined by epi-fluorescence using alexa fluor 488 (excitation 495 nm and emission 515 nm). Positive control leaf and root samples were treated with 100 µM sodium nitroprusside and negative control samples were pre-incubated with 100 µM cPTIO for 2 hours. Corrected total cell fluorescence (CTCF) analysis of the microscopic images was performed using ImageJ software.

### Biochemical assays

#### Malondialdehyde (MDA) content

MDA content in plants is a direct measure of lipid peroxidation by RONS. MDA content was determined as in^63^. Tomato seedling (leaves and roots) samples (0.5 gm) were powdered using liquid N_2_ and were homogenized with 4 ml of 20% Trichloroacetic acid containing 0.5% Thiobarbituric acid. The homogenized samples were incubated at 95 °C in a water bath for 15 min. Samples were immediately transferred to an ice bath for cooling and centrifuged at 10,000 × g for 10 min. The absorbance of 1 ml supernatant was measured by a spectrophotometer at 532 nm.

#### Proline content

Free proline content in plants was measured by the procedure reported earlier^63^. Leaf and root samples (0.5 gm) were ground into a fine powder using liquid N_2_ and were mixed with 2 ml of 3% sulphosalicylic acid in a tube. Homogenized samples were centrifuged at 10,000 × g for 5 min. Acetic acid and ninhydrin reagent (500 μl each) were mixed with supernatant, placed in a boiling water bath for 45 min, and then immediately transferred in ice. An equal amount of toluene was added to each sample, which was then vortexed. The optical density of the upper layer (toluene) was measured at 520 nm by a spectrophotometer.

#### Chlorophyll content

The chlorophyll content was measured by DMSO protocol^63^. Tomato leaves (75 mg) were cut into uniform disc-shaped samples and submerged in 10 ml of dimethylsulphoxide. The tubes were incubated at 65 °C for 4 h. The concentration of chlorophyll a, chlorophyll b, and total chlorophyll were calculated by measuring the absorbance at 663 and 645 nm.

#### Ascorbate content

Ascorbic acid content in tomato leaves and roots was measured colorimetrically using a vitamin C assay Kit (Elabscience, USA). This assay kit is based on the reduction of Fe^3+^ into Fe^2+^ by ascorbic acid and the color developing reaction of Fe^2+^ with phenanthroline. The amount of color production is directly proportional to the amount of ascorbic acid in plants and was measured at 536 nm.

### qPCR for measuring gene expression

Gene expression studies of PR genes, antioxidant enzymes, and hormone synthesis pathway enzymes (Supplemental Table 1) were executed through quantitative PCR (qPCR). The total RNA was isolated from the shoots and roots of tomato seedlings using RNAiso Plus (TAKARA BIO INC, Japan) and converted into cDNA using oligo dT primer by ReverTra Ace® qPCR RT Master Mix with gDNA Remover kit (Toyobo Co. Ltd., Japan). For qPCR, 20-µl reaction mixtures were prepared including 2x SYBR Green master mix (iQ™ SYBR® Green supermix BioRad), 0.3 µM of each primer, 1 µl of cDNA, and DNAse/RNase free water. The qPCR was performed in 96 well plates for 40 cycles at PCR cycle conditions (95 °C for 10 s, 58 °C for 20 s, and 72 °C for 20 s) by CFX96™ Real-Time System (BioRad) thermocycler. The 18 S rRNA gene was used as an endogenous control. All the sample reactions were performed in triplicates two times.

### Liquid chromatography-mass spectrometry analysis of SA and JA

Endogenous plant hormone levels in both treated and control tomato seedlings were analyzed by liquid chromatography-mass spectrometry (LC-MS). The plant samples extracted for hormone LC-MS analysis were prepared by manual protocol^64^. The LC-MS analysis of JA and SA in plant extracts was performed at the National Instrumentation Center for Environmental Management (NICEM), Seoul National University (SNU), Seoul, South Korea. Quantitation was performed with different concentrations of JA and SA.

### Statistical analysis

For each experiment, leave and root samples were randomly collected from 10 plants. All the data obtained have mean values ± SE. Measurements were performed on three replicates for each treatment (n = 3). The data was statistically analyzed by t-Student test to compare the differences between the means using the least significant differences at p < 0.05, p < 0.01, and p < 0.001.

## Supplementary information

Supplementary Data
