# Supplementary material for: Cold Atmospheric Plasma-Activated Water Irrigation Induces Defense Hormone and Gene expression in Tomato seedlings
Source: Sci Rep. 2019 Nov 6;9:16080. doi: 10.1038/s41598-019-52646-z (PMC6834632; doi:10.1038/s41598-019-52646-z)
Supplement: Supplementary file 1 — Supplementary Data [file 41598_2019_52646_MOESM1_ESM.pdf]

Cold Atmospheric Plasma-Activated Water Irrigation Induces Defense Hormone and Gene expression in Tomato seedlings

Bhawana Adhikari[1+], Manish Adhikari[1+*], Bhagirath Ghimire[1], Gyungsoon Park[1], and Eun Ha Choi[1*]

[1]Plasma Bioscience Research Center, Applied Plasma Medicine Center, Department of Electrical and Biological Physics, Kwangwoon University, Seoul, South Korea

*Corresponding author mail: ehchoi@kw.ac.kr;

Co-corresponding author mail: manishadhikari85@gmail.com

+ These authors contributed equally to this work and are joint first authors.

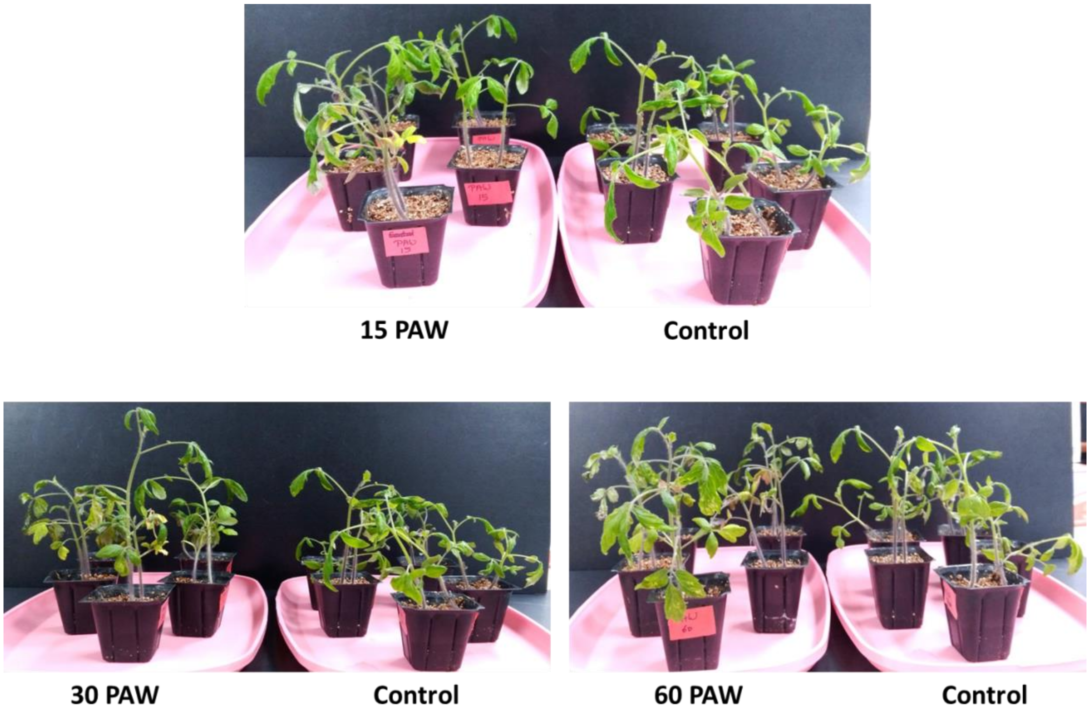

Supplementary figure S1: Growth status of five weeks old tomato plants: Control growth versus PAW treated plants.

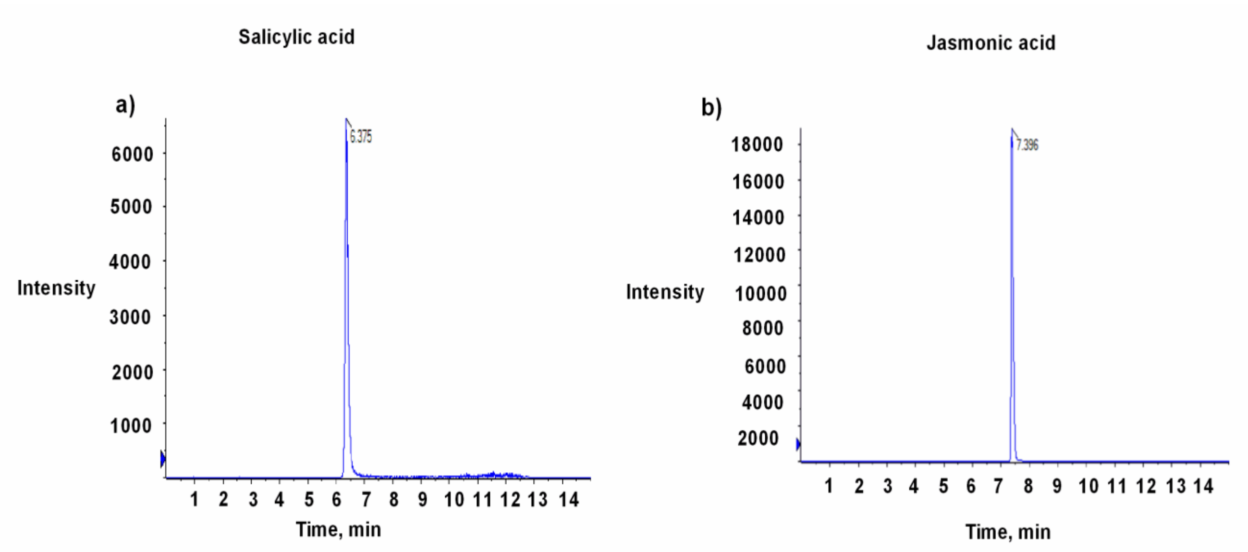

Supplementary figure S2: LC-MS analysis of SA and JA content. (a) SA peak in the chromatogram (RT: 6.36 min) (b) JA peak in the chromatogram (RT: 7.41 min) in 15PAW roots.

Supplementary table 1: List of primers used for gene expression studies.

**Gene**	**Sequence (5'->3')**	**Sequence (5'->3')**	**Length**
Allene oxidase synthase (*aos*)	CGAAACTCCCAGCCCAAAAAG	Plus	21
	ACCTGGTGGCATGTTCGTTC	Minus	20
12-oxophytodienoate reductase (*opr1*)	TGGCACCATTAACAAGGCAAAG	Plus	22
	GTTTCCAAGCCTCCACTTGC	Minus	20
Phenylalanine ammonia lyase (*pal*)	AAAGTGCAAGGGCTGGTGTGA	Plus	21
	TGTGGCAAAGTATGAGATGATTC	Minus	23
Catalase (*cat*)	GTGTACAGACTCCAGTCATTG	Plus	21
	TCTCCTGGATATGGGACTTAG	Minus	21
Mitogen-activated protein kinase 3 (*mapk3*)	ACCTCCTATCATGCCTATTGG	Plus	21
	AGGGCGGAGGAATCACATCT	Minus	20
Superoxide dismutase [Cu-Zn] 1(*sod*)	GACCACATTACAATCCTGCTG	Plus	21
	CAATGATGGACTGTGGACCAG	Minus	21
Glutathione-S-transferase (*gst*)	GTGCCTGTTATTGTTCATGATGG	Plus	23
	CACCGATTCAACTCCCTCTG	Minus	20
β-1,3-Glucanase (*glu*)	AGGAACGATGTTAGATGGTTTACT	Plus	24
	ATCCATCGCAGCATAAACAG	Minus	20
Chitinase 3 acidic (PR3)	CAATTCGTTTCCAGGTTTTG	Plus	20
	ACTTTCCGCTGCAGTATTTG	Minus	20
Glyceraldehyde 3-phosphate dehydrogenase (*gapdh*) Endogenous gene	ATGATGTTGAACTCGTCGCAG	Plus	21
	ACAGTAACAGCCTTCTCACCA	Minus	21
